# Hysterectomy: Current Methods and Alternatives

**DOI:** 10.1155/2010/705073

**Published:** 2011-05-23

**Authors:** Liselotte Mettler, Harry Reich, Limin Feng, Shailesh Puntambekar, Adolf Gallinat, Michael Stark

**Affiliations:** ^1^Department of Obstetrics and Gynecology, University Hospitals Schleswig-Holstein, Campus Kiel, 24105 Kiel, Germany; ^2^Wilkes-Barre General Hospital, Wilkes-Barre, PA 18764-0001, USA; ^3^Capital Medical University, Beijing 10073, China; ^4^Galaxy Laparoscopy Institute, Pune 411004, India; ^5^Tagesklinik Altonaer strasse, 20357 Hamburg, Germany; ^6^New European Surgical Academy, 10117 Berlin, Germany

Hysterectomies performed in the field of obstetrics and gynaecology until the 19th century always had a lethal end. In the 20th century they were performed quite successful concerning survival but perhaps too frequently, whereas the 21st century has witnessed a steep decline in hysterectomy numbers. It is, therefore, an opportune time to review the indications for hysterectomies and hysterectomy techniques as well as the present and future status of this surgical procedure. 

There is a widespread consensus that hysterectomies are primarily to be performed in cancer cases and obstetrical chaos situations even though minimal invasive surgical (MIS) technologies have made the procedure more patient friendly than the classical abdominal opening. Today, minimally invasive hysterectomies are performed as frequently as vaginal hysterectomies, and the vaginal approach is still the first choice if the correct indications are given. It is rarely necessary to open the abdomen; this procedure has been replaced by laparoscopic surgery with multiple- and single-port entries. Laparoscopic and robotic-assisted laparoscopic surgery can also be indicated for hysterectomies in selected patients with gynaecological cancers.

For women of reproductive age, laparoscopic myomectomies and numerous other uterine-preserving techniques are applied in a first treatment step of meno-metrorrhagia, uterine adenomyosis, and submucous myoma. These interventions are only followed by a hysterectomy if the pathology prevails.

We have selected six papers to reflect the present status of hysterectomies and alternate techniques preserving the uterus. In the time of organ preserving minimal invasive surgery in splendid cooperation with imaging technologies and early recognition of the disease, the number of hysterectomies definitely decreases and will hopefully one day be only used in cases of malignancy. Even malignancy treatment, of course, totally depends on early recognition and possibly can also be treated by different modalities than resection in the future.

*Liselotte Mettler* *Liselotte Mettler* *Harry Reich* *Harry Reich* *Limin Feng* *Limin Feng* *Shailesh Puntambekar* *Shailesh Puntambekar* *Adolf Gallinat* *Adolf Gallinat* *Michael Stark* *Michael Stark*

